# Emerging trends on the mechanism of pelvic organ prolapse from 1997 to 2022: visualization and bibliometric analysis

**DOI:** 10.3389/fmed.2023.1158815

**Published:** 2023-06-07

**Authors:** Xia Yu, Wenyi Lin, Xuemei Zheng, Li He, Zhenglin Yang, Yonghong Lin

**Affiliations:** ^1^Department of Clinical Laboratory, Chengdu Women’s and Children’s Central Hospital, Sichuan Provincial People’s Hospital, School of Medicine, University of Electronic Science and Technology of China, Chengdu, Sichuan, China; ^2^Department of Medical Pathology, Chengdu Women’s and Children’s Central Hospital, School of Medicine, University of Electronic Science and Technology of China, Chengdu, Sichuan, China; ^3^School of Medicine, University of Electronic Science and Technology of China, Chengdu, Sichuan, China; ^4^Department of Obstetrics and Gynecology, Chengdu Women’s and Children’s Central Hospital, School of Medicine, University of Electronic Science and Technology of China, Chengdu, Sichuan, China; ^5^Sichuan Provincial Key Laboratory for Human Disease Gene Study, Institute of Laboratory Medicine, Sichuan Provincial People’s Hospital, University of Electronic Science and Technology of China, Chengdu, China

**Keywords:** pelvic organ prolapse, mechanism, CiteSpace, bibliometric analysis, extracellular matrix remodeling, oxidative stress

## Abstract

**Objective:**

At present, there is no feature description of the mechanism of pelvic organ prolapse (POP) in the literature. This study aimed to map the emerging trends regarding the mechanism of POP from inception to 2022 by bibliometric analysis and to analyze its research hotspots and frontiers.

**Methods:**

We downloaded pertinent publications from inception to 2022 from the Web of Science Core Collection (WoSCC) on 30 June 2022. The data were then examined using the Bibliometrix program in R (Version 4.1.0), CiteSpace software, the Online Analysis Platform of Literature Metrology (https://bibliometric.com), and a bibliometrix online interface.

**Results:**

A total of 290 qualified records on the mechanism of POP were identified and included in the analysis. The most productive journal was International Urogynecology Journal. Bump RC and Olsen AL were the most cited authors. Extracellular matrix, collagen, apoptosis, elastin, oxidative stress, gene expression, matrix metalloproteinase, and tissue engineering were among the 25 most relevant terms. According to the analysis of trending topics, tissue engineering has become a new research hotspot.

**Conclusion:**

Extracellular matrix remodeling, oxidative stress and apoptosis are the three main directions for studying the mechanism of POP. In addition, tissue engineering has become a new research hotspot. In the future, in-depth research on the interaction between different mechanisms will be carried out, and attempts will be made to combine biomimetic materials and seed cells to achieve the regeneration and reconstruction of POP-related organs.

## Introduction

Pelvic organ prolapse (POP) is characterized by the herniation of pelvic organs, like the uterus, bladder, small bowel, and rectum, into the vaginal cavity. Although POP is not fatal, it has a significant impact on patient quality of life (1). The incidence of POP increases with age (2). And the POP population is expected to grow by 46% to 4.9 million by 2050. According to studies, women have a 12–19% lifetime risk of requiring POP surgery; however, the therapeutic effect is limited, with more than 10% of patients requiring reoperation after 5 years (3). In patients who have been treated with mesh, the recurrence rate increases, and there are more complications (4). Consequently, it is important to elucidate the mechanism of POP to find new treatment methods.

Many authors around the world have published studies on the mechanism of POP, especially in recent years, and with the development of omics technology, the study of POP has been greatly promoted (5–7). Thus, it is necessary to collect information from related publications to aid researchers in their analysis of the extensive body of literature on this subject and to quickly grasp the overall direction of this research and conduct research in this field with little to no prior knowledge.

Bibliometric analysis is a method that has been widely used in the medical field to analyze large amounts of heterogeneous literature (8). To conduct this study, we made use of CiteSpace (9) to find and visualize information from the Web of Science Core Collection (WoSCC) from diverse perspectives, including various countries, institutions, co-cited authors, and co-cited references. Moreover, the “bibliometrix” R package, and “biblioshiny” were also used to create trending topics and identify research hotspots (10).

To our knowledge, bibliometric analysis has not yet been applied to the literature regarding the mechanism of POP. In this study, we attempted to direct future studies by illustrating the evolution of the literature on the mechanism of POP and discussing future directions for this area of study.

## Materials and methods

### Data sources and search strategies

To lessen bias associated with database updates, relevant publications were searched for in the WoSCC Science Citation Index Expanded (SCI-Expanded) and Social Sciences Citation Index (SSCI). The applied search technique was as follows: TS = (“Pelvic organ prolapse” or “pelvic organ prolapse” or “pelvic organ prolapses”) AND ALL = (“mechanism” or “mechanisms” or “pathogenesis” or “proliferation” or “apoptosis” or “Mechanical stress” or “cytoskeleton” or “ECM”) NOT AB = (“systematic reviews” or “meta-analyses”) NOT TI = (“guideline” or “recommendation” or “consensus” or “case report” or “meta” or “review” or “mesh”). Document Type was set to include “Articles” only from inception to the present (last search date on 30 June 2022). Following the primary data search, two researchers (LH and XZ) independently reviewed each manuscript to ensure that it was pertinent to the topic of this study.

### Data analysis and data visualization

The WoSCC data were downloaded as a tab-delimited text file and transported into Bibliometrics’ Online Analysis Platform^1^, where the “Total volume” option was used for publication trend analysis of various years and the “National total” option was used for publication quantity analysis of countries.

From the WoSCC database, complete records and cited references for these articles were obtained. Using the following parameters, the TXT format files were then imported into CiteSpace program V6.1R2 (64-bits) Basic (Drexel University, Philadelphia, PA, USA). The time span (January 1997–June 2022), years per slice (1), links (strength: cosine, scope: within slices), selection criteria (g-index: *k* = 25, Top *N* = 50, Top N% = 10%, maximum number of selected items per slice = 100), pruning (Pathfinder, Pruning sliced networks), and all of the other parameters were left at their default settings. The node type parameter area was set as follows: “Country” was chosen for intercountry analysis, “Institution” was chosen for interinstitutional analysis, “Cited-author” was chosen for coauthorship network analysis, and “References” was chosen for document co-citation analysis.

The Bibliometrix package in R (Version 4.1.0) and a web interface for bibliometrix, “biblioshiny,” were used to obtain the most often cited documents and the most frequent word and trend topics using the.TXT format data already saved.

## Results

### Quantity and trend analysis of publications

**FIGURE 1:**
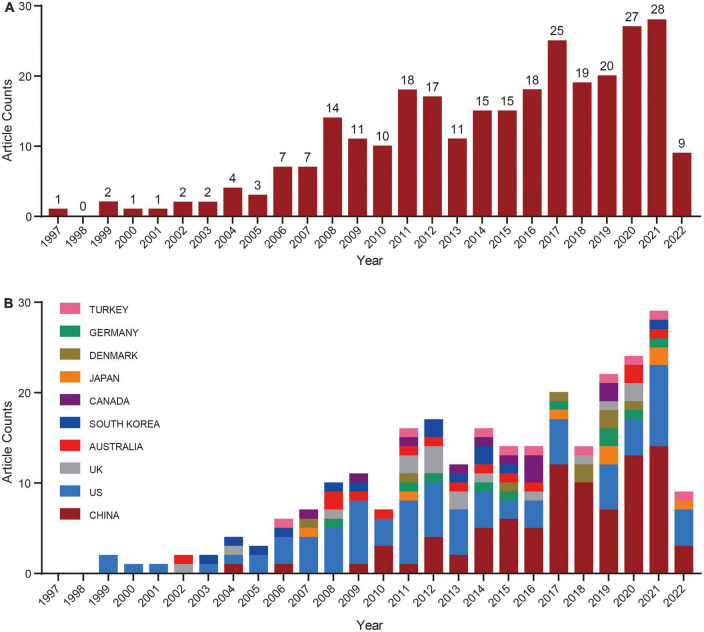
The number of publications on the mechanism of pelvic organ prolapse and the growth trend from the beginning to June 2022. **(A)** Number and growth trends of annual research publications. **(B)** Number and growth trends in annual publications in the top 10 countries. Bar chart, the number of online articles per year.

With the aforementioned parameters, 290 articles were eligible for inclusion. The Bibliometrics Online Analysis Platform(see text footnote 1) was used to determine the annual number of papers published (Figure 1A). From inception to the present (1997–2022), the number of articles published each year was less than 50. There was a significant increase in 2006, which peaked in 2021, and during this time, the mechanism of POP became a focal point of this research field.

In addition, further analysis of publications from different countries was performed to identify countries conducting research in this field. As shown in Figure 1B, we used a bar chart to show the top 10 countries for the total number of articles published from 1997 to 2022. The USA was noted as a pioneer in the field based on publications, which are currently gradually rising. Additionally, we discovered that starting in 2014, China published more articles every year than the United States.

### Journal analysis

The 290 papers that made up the current analysis were revealed by the WoSCC search to have appeared in 128 distinct journals over the course of the last 25 years, starting in 1997. The impact of the journals was examined using bibliometric online analysis. Supplementary Table 1, which lists the 10 most referenced journals, shows that five of the publishers were from the United States, while four were from the Netherlands, Germany, Greece, and the United Kingdom. The International Urogynecology Journal had the most papers published and the most citations overall (84), with an impact factor (IF) of 1.932. The American Journal of Pathology had an IF of 5.770 and the greatest average number of citations (17.67).

### Analysis of intercountry/regional and interinstitutional cooperation

To identify research institutions and interinstitutional cooperation in research into the mechanism of POP, intercountry/regional and interinstitutional analyses were performed using CiteSpace, and we retained 290 published articles for inclusion in the final analysis after removing duplicate entries.

**FIGURE 2:**
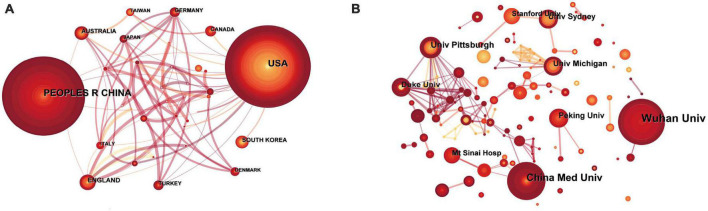
A collaboration to study the mechanism of POP was visualized using CiteSpace. **(A)** Intercountry cooperation. **(B)** Interinstitutional cooperation. Each circle represents a country/institution. The size of the circle is positively correlated with the number of articles published by the country/institution, and the link between the two circles indicates the cooperation of the two institutions on the same article. Line thickness is positively correlated with the frequency of collaboration.

The results of regional cooperation among countries show that 45 countries have established partnerships, and there are 72 links between them. The United States and China have the highest number of publications in this area. However, China’s international cooperation is not as good as that of the United States (Figure 2A).

Figure 2B (threshold = 6) displays the rankings of the nine most productive institutions. The size of the concentric circles shows the number of publications; thus, institutions with more publications typically have larger concentric circles. A relationship between two institutions indicates that they have coauthored publications. The lines’ boldness denotes how effectively they cooperated. In all, 345 nodes and 489 linkages were found after an investigation of partnerships between the various institutions. The majority of the total was made up of institutions in China and the United States. Wuhan University in China was the most productive (article number = 17). China Med University (article number = 14) and the University of Pittsburgh (article number = 8) were the second and third most productive institutions, respectively, in China and the United States, followed by Duke University, Peking University, the University of Sydney, and the University of Michigan (article number = 7 for each institution).

### Author and document cocitation analysis

**FIGURE 3:**
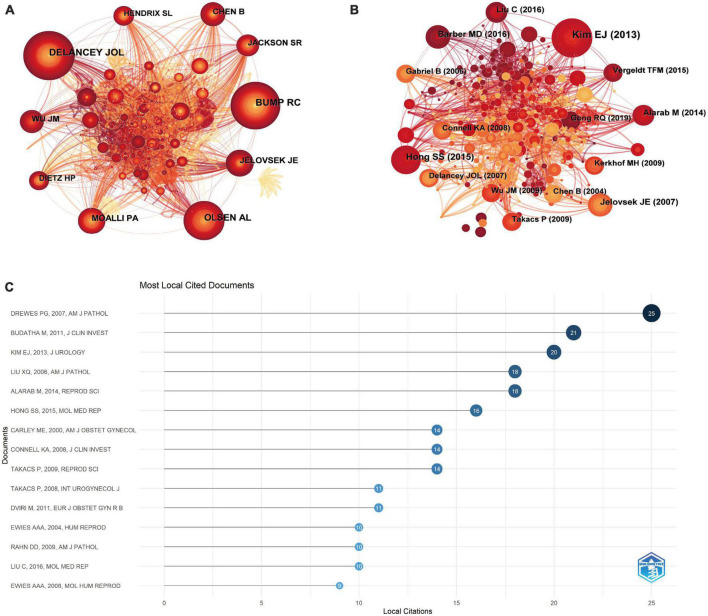
Analysis of co-cited authors and co-cited articles. **(A)** Co-cited author in the field of research on the mechanism of POP. Each circle represents an author. The size of the circle is positively correlated with the number of citations by the author, and the link between the two circles represents the collaboration of two authors on the same article. Line thickness is positively correlated with the frequency of collaboration. **(B)** Cocitation of literature on the mechanism of POP. Each circle represents a reference. The size of the circle is positively correlated with the frequency of citations, and the link between the two circles represents two references cited in the same article. The figure shows the year and first author of the top 15 most cited publications. **(C)** Most local cited documents on the mechanism of POP.

Cocitation analysis can show the direction of current research on the POP molecular mechanism. The 10 most cited authors and 15 most cited references were identified through cited author and cited reference analyses, which can offer crucial insights (Figures 3A, B). When two (or more) writers are simultaneously referenced in one or more subsequent articles, a cocitation link between the authors is established. By examining the co-cited networks of the authors, the strength of which shows the authors’ level of participation, we obtain a clear image of the key authors and their contributions to a certain topic. The 10 most referenced authors and 15 most cited references on the mechanism of POP were found after using CiteSpace to examine the 290 original papers and 626 genuine and distinct references that were gathered from them. The author cocitation analysis produced 626 nodes and 3309 linkages. The thickness of the lines between two nodes represents the frequency between two authors of being co-cited, and the node size is positively correlated with the cited counts of the authors. Figure 3A displays the 10 researchers in this field who have received the most citations. The most frequently referenced author in 1999 was Delancey JOL, who received 90 citations. The second most frequently mentioned author in 1997, Bump RC from the Duke University Medical Center, received 87 citations. Olsen AL, a researcher at Oregon Health Sciences University, was the third most cited author of 2002 with 72 citations. Figure 3A also displays the other seven significant research teams (Jelovsek JE from the Duke University Medical Center, Moalli PA from the University of Pittsburgh, Chen B from the Stanford University School of Medicine, Wu JM from the University of North Carolina, Jackson SR from the Oxford University Hospital, Hendrix SL from the Wayne State University School of Medicine, and Dietz HP from University of Sydney).

In Figure 3B, based on the document cocitation analysis, the 15 most-cited publications are displayed with the year and first author. While the thickness of the lines connecting each pair of nodes indicates the co-occurrence of citations, the size of the circle is positively connected with citation frequency. Supplementary Table 2 contains information on these 15 articles. In addition, to obtain more convincing articles to illustrate the research trend, we also used R software and the bibliometrix codes. The 15 most commonly cited documents are shown in Figure 3C and Supplementary Table 3.

Given that referenced research is frequently conducted to support an author’s concept, a high frequency of citations for an article would indicate that the reference has made a significant contribution to the area and has received peer acknowledgment that is based on solid evidence. Interestingly, oxidative damage, apoptosis, and disruption of extracellular matrix (ECM) equilibrium were the main topics of research in the 15 most-cited studies and the 15 most local cited documents (11–16). Among these, on the one hand, the highest-ranking article of the most-cited studies published in the Journal of Urology in 2013 (13) showed in correlation analyses that the percentage of immune-positive cells with 8-OHdG or 4-hydroxy-2-non-enal was significantly positively correlated with markers of mitochondrial apoptosis. This result was consistent with the second highest cocitation, released by Molecular Medicine Reports (15). On the other hand, employing *Fbln5^–/–^* mice (17), which, along with *Loxl1^–/–^* mice, are another mutant mouse model of POP (18), the most local cited articles have demonstrated that disrupted elastic fiber homeostasis is a major event in the pathogenesis of POP.

### Clustered network in coanalysis

**FIGURE 4:**
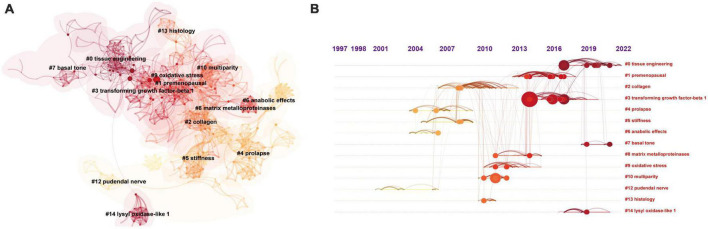
Cluster network analysis of reference cocitation. **(A)** A cluster network of cocitation status for references and cited articles via CiteSpace. Displays the top 15 largest clusters of the referenced article. **(B)** Timeline view of the top 15 clusters of the article is cited.

To delve deeper into the co-cited articles, we performed a cluster network analysis. According to the logic of homogeneity analysis, two publications tend to be homogeneous if they have many references, so we grouped the 290 articles into different clusters. After filtering by selecting the “Show the Largest K Clusters” node (*K* = 15), (which could explain why the number of clusters displayed is not continuous), 14 major clusters generated from the cocitation networks of 626 references cited by 290 publications were identified. Cluster labels are notable noun phrases extracted from keywords using the least square filtering (LSR) algorithm, including #0 tissue engineering, #1 premenopausal, #2 collagen, #3 transforming growth factor-beta 2, #4 prolapse, #5 stiffness, #6 anabolic effects, #7 basal tone, #8 matrix metalloproteinases, #9 oxidative stress, #10 multiparity, #12 pudendal nerve, #13 histology, and #14 lysyl oxidase-like 1 (Figure 4A). The number of articles per cluster that are included is negatively correlated with the number of cluster tags.

Figure 4B shows various timeline representations of co-cited studies to more clearly display all the cited literature. The bold timeline shows that there was much discussion about the cluster during this time. Some of the important studies with a high frequency of citations are represented by citation tree rings of various sizes on the timeline.

Collagen has been a major issue in research on the mechanism of POP since 2006, reaching its peak in 2008. Since then, research has also focused on matrix metalloproteinases (MMPs) and transforming growth factor-beta 1 (TNF-1) in relation to collagen synthesis. MMP studies first surfaced in 2011 and strongly resurged in 2014. In 2013, TNF-1 research was still in its infancy but quickly gained popularity. In 2011, oxidative stress (OS) also became another research hotspot. However, this hotspot lasted only until 2015 and ended. On the other hand, tissue engineering research has continued since its emergence in 2016.

### Research trend analysis of keywords

**FIGURE 5:**
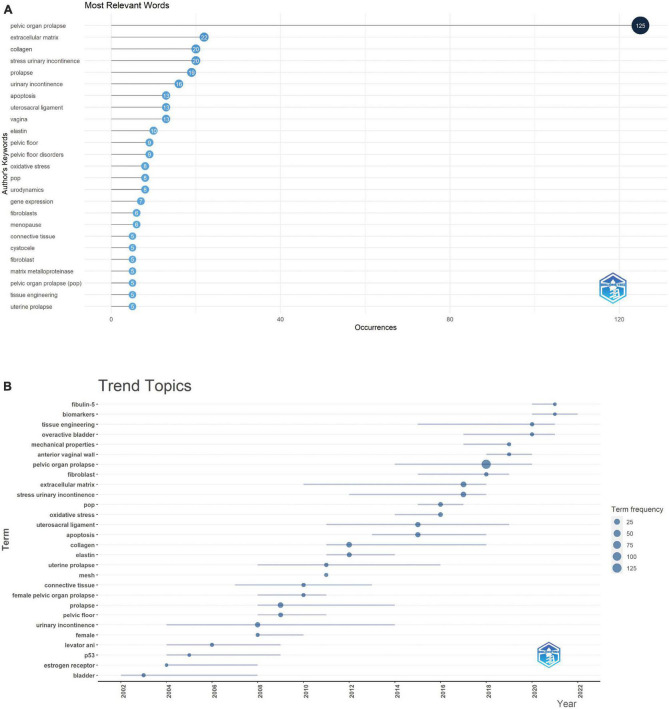
Analysis of author keywords. **(A)** The most relevant words in the top 25. **(B)** Trend topics are shown.

Author keywords were used to acquire a quick glimpse of the most relevant words and trend topics (word minimum frequency = 3; number of words per year = 2) (Figures 5A, B). As shown in Figure 5A, the 25 most relevant words included extracellular matrix, collagen, apoptosis, elastin, oxidative stress, gene expression, matrix metalloproteinase, and tissue engineering. According to the analysis of trend topics, tissue engineering has become a new research hotspot.

## Discussion

The present study provides the first visual analysis of citation patterns in research articles on the mechanisms of POP from 1997 to 2022. Three analytical tools were employed to obtain a comprehensive understanding of the field from various perspectives. The number of published articles on the subject has increased significantly since 2006, with nearly 30 articles per year being published by 2021. Inter-country, inter-institutional, journal, co-cited authors and references, and keyword analyses were conducted to offer a systematic view of the field over the past 25 years. To facilitate a better understanding of the most frequently cited papers, the contents of the 15 most locally cited articles, which have played a crucial role in the development of the field, were discussed. The research content was classified into three categories, namely, ECM remodeling, OS, and apoptosis. Cluster labels are notable noun phrases extracted from keywords, showing that tissue engineering is an important category, so we also discussed the study of tissue engineering articles in POP.

### Extracellular matrix remodeling

In the course of examining 15 articles, it was discovered that 11 of them focused on ECM remodeling in relation to POP, with particular emphasis on the effects of elastic fiber and collagen. Notably, the earliest article among the 15 was published in 2000. Carley et al. discovered that certain individuals may be predisposed to POP due to inherent differences in their connective tissue. Notably, women with Marfan syndrome or Ehlers-Danlos syndrome exhibit a higher incidence of POP, providing evidence that connective tissue disorders are a contributing factor to the condition (19). ECM constitutes the major component of connective tissue, with elastic fibers playing a crucial role in imparting elasticity to the tissue (20). Liu et al. conducted a study on mutant mice lacking LOXL1 and found that defects in elastic fibers were the main cause of complex and severe pelvic floor diseases. In a related study published in the American Journal of Pathology in 2007, researchers examined pelvic organ support in Fbln5^–/–^ mice and concluded that the synthesis and assembly of elastic fibers were critical for the recovery of pelvic organ support after vaginal delivery. The study also found that disordered elastic fiber homeostasis played a primary role in the development of POP in mice (17). The research conducted by Rahn et al. was motivated by the Fbln5^–/–^ and Loxl1^–/–^ mouse models of elastic fiber disorder, which served as a source of inspiration for exploring other matrix proteins involved in elastic fiber assembly or degradation and associated with POP. Through their investigation, they identified the potential involvement of fibulin-3 in pelvic organ support, as they examined the gross and ultrastructural changes in the vaginal muscularis of mice lacking fibulin-3 (Fbln3^–/–^) (21). The preservation of collagen content in the ECM is dependent on ECM-remodeling proteins. An investigation by Connell et al. analyzed the expression of HOXA11, collagen type I, collagen type III, MMP2, and MMP9 in the uterosacral ligaments (USLs) of women with and without POP. The results highlighted the importance of HOXA11 in the development of USL and suggested that changes in a signaling pathway involving HOXA11, collagen type III, and MMP2 may weaken connective tissue in women with POP (11). In 2011, Budatha et al. published findings indicating that MMP-9 levels were markedly increased in vaginal tissue samples collected from pre- and postmenopausal women with POP. The authors emphasized the pivotal role of fibulin-5, which is indispensable for elastic fiber assembly, in controlling MMP-9 activity to maintain the structural integrity of the vaginal wall and prevent the development of POP (22). Correspondingly, Dviri et al. observed that women with POP manifested elevated stromal (extracellular) expression of MMPs-1 and -9 in vaginal and USL biopsies relative to controls. Furthermore, biopsies from individuals with POP evinced augmented intracellular expression of both MMPs in fibroblasts (23). In a subsequent study, Alarab et al. discovered that patients with POP exhibited increased gelatinase activity of MMP-2 and heightened expression of the 58-kDa isoform of a disintegrin and metalloproteinase with thrombospondin motifs [ADAMTS]-2. Conversely, the expression of TIMP-1–4 genes and TIMP-1 protein were significantly decreased, while the protein expression of MMP-12 (both pro and active forms) was significantly elevated (14). The alterations in the ECM’s composition are believed to be the cause of the excessive expression of steroid receptors in the prolapsed principal ligament. In a study conducted by Ewies et al. the expression of gonadal steroid receptors in the cardinal ligaments of prolapsed uteri was compared to that of non-prolapsed controls. The results showed a significant increase in the expression levels of estrogen receptor alpha (ERalpha), ERbeta, progesterone receptor, and androgen receptor in the prolapsed major ligaments. The researchers suggested that these changes may be linked to the tissue stretching trauma process that occurs during vaginal delivery. The study findings indicated a noteworthy elevation in the levels of ERalpha, ERbeta, progesterone receptor, and androgen receptor in the prolapsed major ligaments. The researchers postulated that these variations might be associated with the process of tissue stretching trauma that arises during vaginal delivery (24). Despite the administration of estradiol, Ewies et al. discovered that the cytoskeleton disorder of fibroblasts was not reversed, nor were the cells protected from the impact of stretching. However, the rate of fibroblast proliferation was significantly elevated, indicating a potential contribution to the healing process (25).

### Oxidative stress and apoptosis

In these 15 articles, 4 reported that OS and apoptosis were closely related to the occurrence of POP. In 2008, Takacs et al. conducted an immunohistochemistry study on vaginal smooth muscle and observed that women with anterior vaginal wall prolapse had a considerably lower proportion of vaginal smooth muscle compared to those without prolapse. Additionally, the rate of apoptosis was higher in the former group (26). Then, the USLs of women with POP and those with normal support were analyzed by Kim et al. to determine the biomarkers of OS and mitochondrial apoptosis. According to their findings, patients with POP exhibited significantly higher levels of 8-OHdG, 4-hydroxy-2-non-enal, TUNEL, cleaved caspase-3, and cytochrome c in their USLs compared to controls. Moreover, significant positive correlations were observed between the proportion of cells expressing 8-OHdG or 4-hydroxy-2-non-enal and indicators of mitochondrial apoptosis (13). The research conducted by Hong et al. revealed that the imposition of mechanical strain on human parametrial ligament fibroblasts led to heightened levels of intracellular ROS, a decline in mitochondrial membrane potential, and an increased incidence of apoptosis. These outcomes may contribute to the pathogenesis of POP (15). The involvement of OS in fiber metabolic disorders is widely acknowledged. In light of this, Liu et al. conducted a study to determine the precise role of OS in collagen metabolism in human USL fibroblasts (hUSLFs). Their findings suggest that OS may contribute to collagen metabolic disorder in hUSLFs in a severity-dependent manner, potentially through the indirect regulation of MMPs, TIMPs, and TGF-β1. These results shed light on the pathophysiology of POP and highlight the importance of understanding the role of OS in metabolic disorders (16).

### Tissue engineering

Tissue engineering is a multidisciplinary discipline that studies morphological biological substitutes for the repair of various tissues or organs after injury, including seed cells, biomaterial scaffolds, dynamic culture conditions, and the *in vivo* physiological environment. To date, a variety of stem cells, primary somatic cells and fine cell lines have been investigated in POP. The use of stem cell bioengineered grafts to repair the damaged vaginas of rhesus monkeys, resulting in ECM recombination of large muscle bundles to form angiogenesis and enhance vaginal mechanical properties, which is a new method for the treatment of POP reported by Ma et al. (27). Furthermore, choosing the right biomaterials is necessary for the successful regeneration of the female reproductive system. Based on the study of POP pathogenesis, tissue engineering complexes with a low foreign body response and a structure similar to that of the ECM are expected to promote tissue integration and regeneration. The construction of a polycarbonate supramolecular polymer with ureidopyrimidinone (UPy)-modified cyclic arginine-glycine-aspartic acid (cRGD) peptide additives was described in a study that was published in 2019. When used for abdominal wall reconstruction in a rat hernia model, the polymer led to the formation of physiological neotissue. Moreover, cRGD bioactivation can also promote neovascularization and the growth of connective tissue into the scaffold (28). Another study indicated that methacrylate gelatin loaded with puerarin had anti-inflammatory activity and inhibitory effects on MMP-2 and MMP-9, which could guide pelvic floor connective tissue regeneration (29).

Moreover, these tissue engineering findings provide new insights into POP therapy. Although the application of tissue engineering in POP has been extensively investigated, its poor outcome has been attributed to its clinical application. The reason has been attributed to low reproducibility and risk of immune rejection of natural materials and decellularized extracellular matrix (dECM); moreover, the conditions in the clinical setting are not as precisely defined as in the laboratory. Future research could carry out in-depth studies of biomimetic materials that can replace tissue structure, which achieve the regeneration and remodeling of POP-related organs by combining seed cells with balanced regenerative ability and appropriate dynamic culture conditions.

The limitations of the current analysis must be taken into account. A single database served as the source for the data. Bibliometric analysis, which is a quantitative evaluation of academic works, can only be applied to works that have been cited and indexed in other works; it cannot be applied to works that have not yet been published or that have appeared in unindexed journals, papers, books, or government reports. To learn more about this research subject in depth and with greater information, we will employ a multimethod approach in future investigations.

## Author contributions

ZY and YL: project development and management data analysis. XY and WL: manuscript writing and statistical analysis. XY and XZ: manuscript revision. LH and XZ: data collection. All authors contributed to the article and approved the submitted version.

## Footnotes

1
https://bibliometric.com
