# Supplementary material for: Emerging trends on the mechanism of pelvic organ prolapse from 1997 to 2022: visualization and bibliometric analysis
Source: Front Med (Lausanne). 2023 Jun 7;10:1158815. doi: 10.3389/fmed.2023.1158815 (PMC10282136; doi:10.3389/fmed.2023.1158815)
Supplement: Supplementary file 1 [file Table_1.DOCX]

Supplementary Material

# Supplementary Figures and Tables

## Supplementary Tables

**Table 1. The top 10 most active journals that published articles in the mechanism of pelvic organ prolapse (sorted by total citation)**

| Rank | Journal Title | Frequency | Total citations | Average citation per paper | Impact factor  (2021) | Country | JCR |
| --- | --- | --- | --- | --- | --- | --- | --- |
| 1 | International Urogynecology Journal | 43 | 84 | 1.95 | 1.932 | Germany | Q4 |
| 2 | American Journal of Pathology | 3 | 53 | 17.67 | 5.77 | United States | Q1 |
| 3 | Reproductive Sciences | 5 | 44 | 8.8 | 2.924 | United States | Q3 |
| 4 | Molecular Medicine Reports | 14 | 43 | 3.07 | 3.423 | Greece | Q3 |
| 5 | Journal of Clinical Investigation | 2 | 35 | 17.5 | 19.456 | United States | Q1 |
| 6 | Neurourology and Urodynamics | 16 | 26 | 1.63 | 2.364 | United States | Q3 |
| 7 | Journal of Urology | 5 | 21 | 4.2 | 7.600 | Netherlands | Q1 |
| 8 | Human Reproduction | 3 | 21 | 7 | 6.353 | United Kingdom | Q1 |
| 9 | European Journal of Obstetrics & Gynecology and Reproductive Biology | 10 | 21 | 2.1 | 2.831 | Netherlands | Q3 |
| 10 | American Journal of Obstetrics and Gynecology | 6 | 18 | 3 | 10.693 | United States | Q1 |

**Table 2. The top 15 high-cited papers in in the mechanism of pelvic organ prolapse during 1997 to 2022**

| Rank | Title | First Author | Journal | Year | Cited Frequency | DOI |
| --- | --- | --- | --- | --- | --- | --- |
| 1 | Involvement of oxidative stress and mitochondrial apoptosis in the pathogenesis of pelvic organ prolapse. | Eun Jae Kim | J Urol | 2013 | 15 | 10.1016/j.juro.2012.09.041 |
| 2 | Oxidative damage to human parametrial ligament fibroblasts induced by mechanical stress. | Hong S | Mol Med Rep | 2015 | 11 | 10.3892/mmr.2015.4115 |
| 3 | Pelvic organ prolapse. | Jelovsek JE | Lancet | 2007 | 9 | 10.1016/S0140-6736(07)60462-0 |
| 4 | Pelvic organ prolapse. | Barber MD | BMJ | 2016 | 9 | 10.1136/bmj.i3853 |
| 5 | Expression of extracellular matrix-remodeling proteins is altered in vaginal tissue of premenopausal women with severe pelvic organ prolapse. | Alarab M | Reprod Sci | 2014 | 8 | 10.1177/1933719113512529 |
| 6 | Collagen metabolic disorder induced by oxidative stress in human uterosacral ligament‑derived fibroblasts: A possible pathophysiological mechanism in pelvic organ prolapse. | Liu C | Mol Med Rep | 2016 | 8 | 10.3892/mmr.2016.4919 |
| 7 | Collagen changes in pelvic support tissues in women with pelvic organ prolapse. | Gong R | Eur J Obstet Gynecol Reprod Biol | 2019 | 7 | 10.1016/j.ejogrb.2019.01.012 |
| 8 | Changes in connective tissue in patients with pelvic organ prolapse--a review of the current literature | Kerkhof MH | Int Urogynecol J Pelvic Floor Dysfunct | 2009 | 7 | 10.1007/s00192-008-0737-1 |
| 9 | HOXA11 is critical for development and maintenance of uterosacral ligaments and deficient in pelvic prolapse. | Connell KA | J Clin Invest | 2008 | 7 | 10.1172/JCI34193 |
| 10 | Elastolytic activity in women with stress urinary incontinence and pelvic organ prolapse. | Chen B | Neurourol Urodyn | 2004 | 7 | 10.1002/nau.20012 |
| 11 | Uterosacral ligament smooth muscle cell apoptosis is increased in women with uterine prolapse. | Takacs P | Reprod Sci | 2009 | 7 | 10.1177/1933719108328611 |
| 12 | Comparison of levator ani muscle defects and function in women with and without pelvic organ prolapse. | DeLancey JO | Obstet Gynecol | 2007 | 7 | 10.1097/01.AOG.0000250901.57095.ba |
| 13 | Increased expression of matrix metalloproteinase 2 in uterosacral ligaments is associated with pelvic organ prolapse | Gabriel B | Int Urogynecol J Pelvic Floor Dysfunct | 2006 | 7 | 10.1007/s00192-005-0045-y |
| 14 | Forecasting the prevalence of pelvic floor disorders in U.S. Women: 2010 to 2050. | Wu JM | Obstet Gynecol | 2009 | 7 | 10.1097/AOG.0b013e3181c2ce96 |
| 15 | Risk factors for pelvic organ prolapse and its recurrence: a systematic review. | Vergeldt TF | Int Urogynecol J | 2015 | 7 | 10.1007/s00192-015-2695-8 |

**Table 3. The top 15 most local cited documents in in the mechanism of pelvic organ prolapse during 1997 to 2022**

| Rank | Document | Title | Year | Local Citations | Global Citations | LC/GC Ratio (%) | Normalized Local Citations | Normalized Global Citations |
| --- | --- | --- | --- | --- | --- | --- | --- | --- |
| 1 | Drewes PG, 2007, Am J Pathol | Pelvic organ prolapse in fibulin-5 knockout mice: pregnancy-induced changes in elastic fiber homeostasis in mouse vagina | 2007 | 25 | 128 | 19.53 | 5.00 | 4.02 |
| 2 | Budatha M, 2011, J Clin Invest | Extracellular matrix proteases contribute to progression of pelvic organ prolapse in mice and humans | 2011 | 21 | 96 | 21.88 | 6.75 | 4.61 |
| 3 | Kim EJ, 2013, J Urology | Involvement of oxidative stress and mitochondrial apoptosis in the pathogenesis of pelvic organ prolapse | 2013 | 20 | 33 | 60.61 | 4.23 | 1.49 |
| 4 | Liu XQ, 2006, Am J Pathol | Failure of elastic fiber homeostasis leads to pelvic floor disorders | 2006 | 18 | 117 | 15.38 | 3.07 | 3.13 |
| 5 | Alarab M, 2014, Reprod Sci | Expression of extracellular matrix-remodeling proteins is altered in vaginal tissue of premenopausal women with severe pelvic organ prolapse | 2014 | 18 | 33 | 54.55 | 6.14 | 2.05 |
| 6 | Hong SS, 2015, Mol Med Rep | Oxidative damage to human parametrial ligament fibroblasts induced by mechanical stress | 2015 | 16 | 30 | 53.33 | 6.00 | 2.51 |
| 7 | Carley ME, 2000, Am J Obstet Gynecol | Urinary incontinence and pelvic organ prolapse in women with Marfan or Ehlers Danlos syndrome | 2000 | 14 | 129 | 10.85 | 1.00 | 1.00 |
| 8 | Connell KA, 2008, J Clin Invest | HOXA11 is critical for development and maintenance of uterosacral ligaments and deficient in pelvic prolapse | 2008 | 14 | 51 | 27.45 | 3.16 | 1.21 |
| 9 | Takacs P, 2009, Reprod Sci | Uterosacral ligament smooth muscle cell apoptosis is increased in women with uterine prolapse | 2009 | 14 | 23 | 60.87 | 3.28 | 0.64 |
| 10 | Takacs P, 2008, Int Urogynecol J Pelvic Floor Dysfunct | Vaginal smooth muscle cell apoptosis is increased in women with pelvic organ prolapse. | 2008 | 11 | 40 | 27.50 | 2.48 | 0.95 |
| 11 | Dviri M, 2011, Eur J Obstet Gynecol Reprod Biol | Increased matrix metalloproteinases-1,-9 in the uterosacral ligaments and vaginal tissue from women with pelvic organ prolapse | 2011 | 11 | 37 | 29.73 | 3.54 | 1.78 |
| 12 | Ewies AA, 2004, Hum Reprod | Changes in gonadal steroid receptors in the cardinal ligaments of prolapsed uteri: immunohistomorphometric data. | 2004 | 10 | 35 | 28.57 | 2.86 | 1.36 |
| 13 | Rahn DD, 2009, Am J Pathol | Failure of pelvic organ support in mice deficient in fibulin-3 | 2009 | 10 | 53 | 18.87 | 2.34 | 1.47 |
| 14 | Liu C, 2016, Mol Med Rep | Collagen metabolic disorder induced by oxidative stress in human uterosacral ligament‑derived fibroblasts: A possible pathophysiological mechanism in pelvic organ prolapse | 2016 | 10 | 27 | 37.04 | 4.29 | 2.02 |
| 15 | Ewies AA, 2008, Mol Hum Reprod | Changes in transcription profile and cytoskeleton morphology in pelvic ligament fibroblasts in response to stretch: the effects of estradiol and levormeloxifene | 2008 | 9 | 27 | 33.33 | 2.03 | 0.64 |
